# Biomarkers of response to PD-1 pathway blockade

**DOI:** 10.1038/s41416-022-01743-4

**Published:** 2022-02-28

**Authors:** Hanxiao Li, P. Anton van der Merwe, Shivan Sivakumar

**Affiliations:** 1 Green Templeton College, University of Oxford, Oxford, UK; 2 Sir William Dunn School of Pathology, University of Oxford, Oxford, UK; 3 Department of Oncology, University of Oxford, Oxford, UK

**Keywords:** Tumour biomarkers, Cancer immunotherapy, Predictive markers

## Abstract

The binding of T cell immune checkpoint proteins programmed death 1 (PD-1) and cytotoxic T-lymphocyte-associated protein 4 (CTLA-4) to their ligands allows immune evasion by tumours. The development of therapeutic antibodies, termed checkpoint inhibitors, that bind these molecules or their ligands, has provided a means to release this brake on the host anti-tumour immune response. However, these drugs are costly, are associated with potentially severe side effects, and only benefit a small subset of patients. It is therefore important to identify biomarkers that discriminate between responders and non-responders. This review discusses the determinants for a successful response to antibodies that bind PD-1 or its ligand PD-L1, dividing them into markers found in the tumour biopsy and those in non-tumour samples. It provides an update on the established predictive biomarkers (tumour PD-L1 expression, tumour mismatch repair deficiency and tumour mutational burden) and assesses the evidence for new potential biomarkers.

## Introduction

**Fig. 1:**
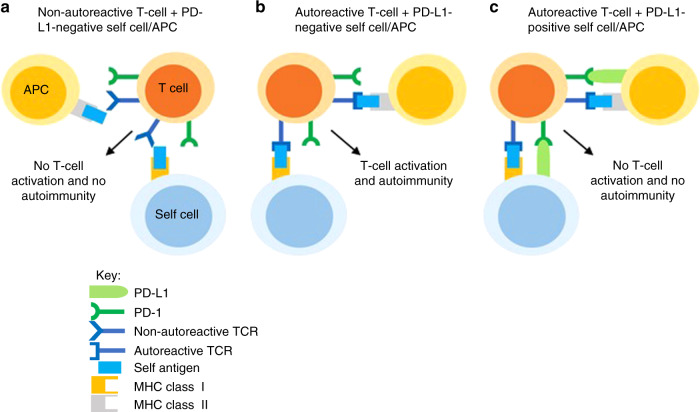
Physiological role of PD-L1. **a** Most T cells are unable to recognise self-antigens, which may be present on the surface of self-cells or APCs. As a result, there is no autoimmunity. **b** Autoreactive T cells can recognise self-antigens and become activated, leading to destruction of the self-cell. **c** PD-L1 expression on self-cells and APCs prevents T cell activation, despite TCR ligation. APC antigen-presenting cell, TCR T cell receptor, MHC major histocompatibility complex.

**Fig. 2:**
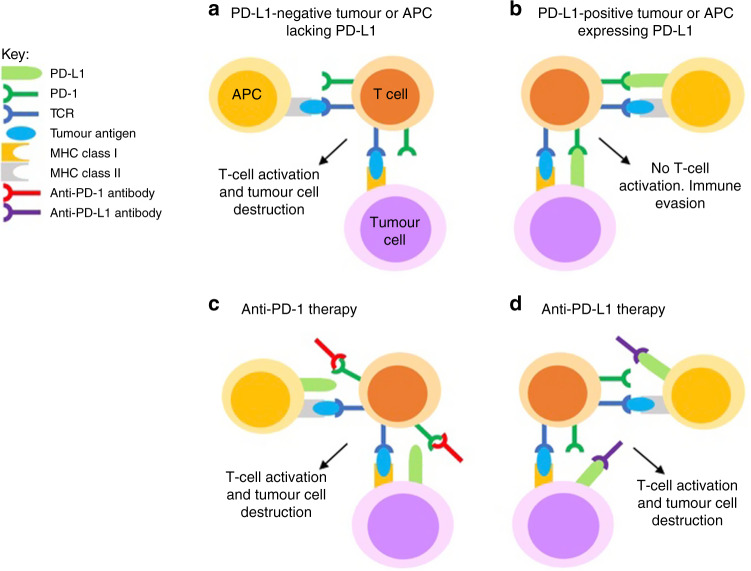
Mechanism of action of anti-PD-1 and anti-PD-L1 antibodies. **a** In a PD-L1 negative tumour, tumour neoantigens presented on the surface of tumour cells or APCs are detected by T cells, which are then activated to destroy the tumour cell. **b** Tumour cell or APC expression of PD-L1 prevents T cell activation and allows immune evasion. **c** Anti-PD-I antibodies can bind to PD-1 on T cells, preventing the interaction between PD-1 and PD-L1 and therefore enabling tumour cell destruction. **d** Anti-PD-L1 antibodies can bind to PD-L1 on tumour cells and APCs, preventing the interaction between PD-1 and PD-L1 and enabling tumour cell destruction.

The host immune system is capable of recognising tumour cells as foreign and destroying them through the action of tumour-antigen-specific T cells. However, this response can be inhibited by the engagement of immune checkpoint proteins expressed on T cells such as programmed death 1 (PD-1) and cytotoxic T-lymphocyte-associated protein 4 (CTLA-4) in lymphoid tissue and in the tumour microenvironment. In a physiological context, the binding of PD-1 and CTLA-4 to programmed death ligand 1 (PD-L1) and B7-1/2, respectively, function to prevent excessive immune responses and autoimmunity (Fig. 1). In the context of a tumour, however, these interactions suppress the host anti-tumour immune response. With the development of immune checkpoint inhibitors (ICIs), monoclonal antibodies that bind to immune checkpoint proteins and their ligands, it has become possible to release this brake on the host anti-tumour immune response and enhance the killing of tumour cells (Fig. 2).

Since the approval of the anti-CTLA-4 antibody ipilimumab in 2011, 6 more checkpoint inhibitors have been approved by the FDA. These consist of the anti-PD-1 antibodies nivolumab, pembrolizumab and cemiplimab, and the anti-PD-L1 antibodies atezolizumab, avelumab and durvalumab (Table 1). However, these drugs are not without side effects. Of particular note is their association with a new class of side effects termed immune-related adverse events (irAEs), which range from fatigue, erythema and hypothyroidism to more serious side effects including gastritis and pneumonitis [1, 2]. In addition to the risk of side effects, checkpoint inhibitor therapy is expensive and only a small subset of patients benefit from treatment. It is therefore important to identify biomarkers that distinguish responders from non-responders.

**Table 1:** MHRA-approved anti-PD-1 and anti-PD-L1 antibodies.

Antibody	Brand name	Approval date	Target	Type	Indication	Company
Pembrolizumab	Keytruda	2014	PD-1	Humanised	Melanoma NSCLC Urothelial carcinoma Classical Hodgkin lymphoma Head and neck SCC Renal cell carcinoma	Merck, Darmstardt, Germany
Nivolumab	Opdivo	2014	PD-1	Human	Melanoma Renal cell carcinoma NSCLC Renal cell carcinoma Urothelial carcinoma Head and neck SCC Classical Hodgkin lymphoma	Bristol Myers Squibb, New York, United States
Atezolizumab	Tecentriq	2016	PD-L1	Humanised	Urothelial carcinoma NSCLC Small cell lung cancer Breast cancer	Genentech, South San Francisco, California, United States
Durvalumab	Imfinzi	2017	PD-L1	Human	NSCLC	AstraZeneca, Cambridge, United Kingdom
Avelumab	Bavencio	2017	PD-L1	Human	Merkel cell cancer Renal cell carcinoma	Merck, Darmstardt, Germany
Cemiplimab	Libtayo	2018	PD-1	Human	Cutaneous SCC	Regeneron, Greenburgh, New York, United States

*NSCLC* non-small-cell lung cancer, *SCC* squamous cell carcinoma.

**Fig. 3:**
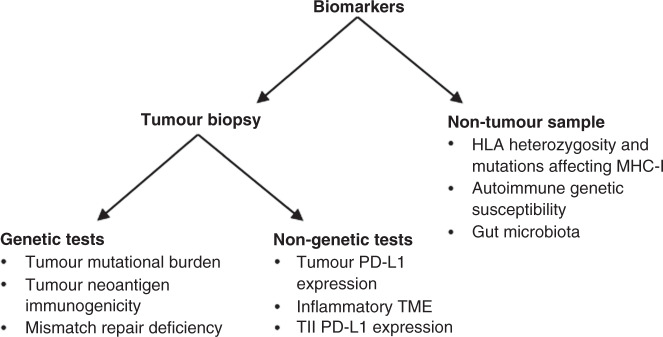
Grouping of biomarkers. Biomarkers are grouped depending on whether they are found in the tumour biopsy or non-tumour samples. Those found in the tumour biopsy are further subdivided into those obtained through genetic tests and non-genetic other tests. TMB tumour mutational burden, MMR mismatch repair, MHC-I major histocompatibility complex class I, TME tumour microenvironment, TII tumour infiltrating immune cells.

CTLA-4 antibodies are no longer widely used, now limited to just a handful of tumours, due to their lower efficacy and more frequent side effects compared to PD-1 pathway inhibitors [3, 4]. Although targeting both pathways has been shown to improve efficacy by a small amount, the rate of grade 3 or 4 adverse events was more than doubled in one study [5]. As a result of the much greater use of PD-1 pathway targeting drugs, this review focuses solely on the biomarkers for anti-PD-1 and anti-PD-L1 antibodies. Currently, the only predictive biomarkers approved for clinical use are tumour PD-L1 expression, tumour mismatch repair deficiency and high tumour mutational burden. However, there is growing evidence for other biomarkers, including the PD-L1 status of tumour infiltrating immune cells and the composition of the host microbiota. This review divides potential biomarkers into those found in the tumour biopsy and those found in non-tumour samples, with tumour biopsy biomarkers being further subdivided into those obtained through genetic tests and those obtained through non-genetic tests such as immunohistochemistry (Fig. 3).

## Biomarkers in the tumour biopsy that are determined by genetic tests

### Tumour mutational burden

Tumour neoantigens generated by non-synonymous mutations can be recognised by CD8^+^ T cells, initiating the host anti-tumour immune response. The presence of a large number of mutations within the tumour DNA, in other words a high tumour mutational burden (TMB), increases the likelihood of neoantigen formation and is therefore associated with a greater CD8^+^ T cell response upon checkpoint inhibition. This is supported by a meta-analysis across patients with 27 tumour types or subtypes showing that TMB is correlated with objective response rate following anti-PD-1 or anti-PD-L1 therapy [6]. A recent study prospectively exploring a retrospective analysis of the phase 2 KEYNOTE-158 study, in which patients with previously treated recurrent or metastatic advanced solid tumours received pembrolizumab, found that high TMB was associated with increased objective response rate [7]. This led to the FDA approval in June 2020 of pembrolizumab in the treatment of unresectable or metastatic solid tumours with a high tumour mutational burden, defined as at least 10 mutations per megabase.

In addition to direct measurement of the TMB itself, studies have shown that individual mutations causing downstream effects on TMB are also predictors of response. For instance, mutations in *POLE* and *POLD1*, which encode DNA polymerases required for proofreading during DNA replication, leading to an increase in replication errors and therefore a greater TMB. These mutations have been found to be associated with a positive response to ICIs across several different cancer types [8].

However, the predictive value of TMB is limited by the presence of intratumoural heterogeneity (ITH); a high ITH indicates that the neoantigen may only be present on a subset of cells and so the immune response generated against the neoantigen may not be effective against the whole tumour. In addition, ITH may result in a lower dosage of each neoantigen, reducing CD8^+^ T cell activation. This provides an explanation for the finding that clonal neoantigens (neoantigens that are present in a large proportion of tumour cells as they were originally derived from a single tumour cell) are enriched in patients who respond well to ICI therapy, whereas sub-clonal mutations are enriched in poor-responders, despite these sub-clonal mutations contributing to an increased TMB [9]. The combination of reduced neoantigen dosage and targeting of only a subset of tumour cells means that it is difficult to establish a single TMB cut-off for response to PD-1 pathway blockade across all tumours and all patients. Single measurements of TMB therefore should not be viewed in isolation but should be considered with multiple tumour biopsies to account for ITH.

A further limitation arises from the techniques used to determine TMB. While original studies linking TMB with response to PD-1 pathway blockade used whole-exome sequencing to determine TMB, this is a costly and labour-intensive technique that would not be feasible in the clinical setting. Instead, targeted gene sequencing is commonly used. The most obvious limitation of this technique is the need to extrapolate the TMB from a small number of genes; one study found that targeting any less than 0.5 Mb of genomic space caused the accuracy of targeted gene sequencing to fall significantly [10]. Another potential limitation is the availability of many different panels which vary in the genes targeted, number of genes tested, and genomic space sequenced. This serves both as a potential source of variation and a barrier to establishing a single standardised technique.

### Tumour neoantigen immunogenicity

It is important to note that only mutations generating neoantigens that are recognised by T cells will contribute to an anti-tumour immune response. In other words, synonymous mutations, or non-synonymous mutations that do not generate immunogenic epitopes, may not stimulate an anti-tumour immune response despite contributing to the TMB. Furthermore, frameshift mutations are likely to result in a more immunogenic neoantigen than a point mutation. By analysing the tumour transcriptome, it is possible to identify immunogenic neoantigens based on their differential agretopicity index, which reflects the difference in the ability of a mutant peptide and its wildtype counterpart to bind to major histocompatibility complex class I (MHC-I). Using this index, it was found that mutations changing the anchor residues (residues that bind to specific pockets on MHC-I to enable MHC-peptide binding) are more likely to be immunogenic [11]. A separate study found that another factor predicting immunogenicity is the similarity of a neoantigen to known pathogenic epitopes [12]. This is supported by the fact that Merkel cell carcinoma and renal cell carcinoma, often associated with Merkel cell polyomavirus and human papilloma virus, respectively, respond better to PD-1 pathway blockade than would be predicted by TMB alone [6]. These studies show that it is possible to predict tumour neoantigen immunogenicity at a genomic and transcriptomic level. However, bioinformatic approaches are not without limitations. Bioinformatics, by nature, involves processing large amounts of data and many putative neoepitopes may be identified based on transcriptomic data alone that may not always correlate with the immunogenic epitopes confirmed using the proteomic assay. To illustrate, the former study mentioned found that only 56% of the putative neoepitopes showed immunogenicity using an IFN-γ ELISpot assay, which fell further to 33% in vivo. Therefore, bioinformatic approaches may be of limited use in the clinical setting, but more useful in the initial stages of identifying putative neoepitopes.

### MMR deficiency

Mismatch repair (MMR) is a DNA repair mechanism used to correct mismatched nucleotides, which may arise from errors during DNA replication or recombination. MMR deficiency leads to hypermutation, which drives neoantigen production, and so MMR deficient tumours produce a stronger anti-tumour response upon PD-1 pathway inhibition. The hypermutation associated with MMR deficiency can be detected as an accumulation of mutations at microsatellites, termed microsatellite instability (MSI). Following a study demonstrating that MMR deficient tumours with increased MSI have a higher response rate to pembrolizumab than MMR proficient tumours [13], pembrolizumab was approved by the FDA for the treatment of advanced solid tumours with the MSI-high or DNA MMR deficiency biomarker in 2017. This was of great significance as it was the first approval to be made based on a pan-tumour biomarker rather than based on tumour type and paved the way for the later approval of high TMB as a pan-tumour biomarker. However, this does not mean that determination of MMR status is useful in all cancers; although MMR deficiency is common in colorectal, gastric, and endometrial cancer, it is less commonly seen in other cancers such as breast cancer and sarcoma and has never been reported in many cancers including acute myeloid leukaemia and nasopharyngeal carcinoma [14]. In these MMR proficient cancers, genetic testing for MMR deficiency is unlikely to yield useful information and instead, assessment of other biomarkers such as tumour PD-L1 expression and TMB may be more useful.

## Biomarkers in the tumour biopsy that are determined by non-genetic tests

### Tumour PD-L1 expression

Interferon-gamma (IFN-γ) released from tumour infiltrating lymphocytes (TILs) induces PD-L1 expression by tumour cells. Therefore, PD-L1 expression may be indicative of T cell infiltration and the presence of a suppressed anti-tumour immune response, and it follows that tumours expressing higher levels of PD-L1 may be subject to a greater immune response upon PD-1 pathway inhibition. Several studies have shown a positive correlation between PD-L1 expression and response to PD-1 pathway blockade [15–17]. In particular, a landmark phase III study in 2016, KEYNOTE-024, showed that non-small-cell lung cancer (NSCLC) patients expressing PD-L1 on over 50% of tumour cells experienced a longer progression-free survival and overall survival after treatment with pembrolizumab compared to platinum-based chemotherapy [18]. This led to the approval of pembrolizumab as a first-line treatment for metastatic NSCLC in patients with over 50% of tumour cells expressing PD-L1. However, there is currently still insufficient evidence to support the clinical use of PD-L1 expression across all cancer types.

In addition, the existing evidence is limited by inconsistencies between studies. The use of different antibodies for PD-L1 and different immunohistochemistry procedures in assessing PD-L1 expression may lead to discrepancies in results; a study testing 3 different antibodies on NSCLC samples found that the antibodies were only moderately concordant in their positive rates [19]. When only antibody assays approved by the FDA were analysed, a systematic review found that while three of the antibodies were interchangeable, SP142-based assays had a low concordance with the other assays [20]. Another point of inconsistency between PD-L1 expression studies is that there is no standardised threshold at which tumours are deemed to be positive or negative for PD-L1, with 1, 5, 10 and 50% being frequently used in the literature. A summary table of phase 3 trials of PD-1 pathway inhibitors which stratified patients by PD-L1 expression demonstrates the effect of different thresholds on treatment benefit and shows a general, although inconsistent, trend of increasing efficacy with higher cut-offs (Table 2). At thresholds above 50%, an even greater benefit can be seen; a retrospective study in patients with non-small-cell lung cancer found that PD-L1 expression levels of 90–100% were associated with an almost doubled overall response rate (60.0% versus 32.7%) and significantly prolonged progression-free survival (14.5 versus 4.1 months) compared to levels of 50–89% in a cohort of 187 patients receiving pembrolizumab [21]. However, the use of very high thresholds clinically may result in the omission of patients who would potentially benefit from therapy.

**Table 2:** Effect of PD-L1 cut-off on outcome in phase 3 trials of PD-1 pathway inhibitors.

Trial name	Antibody assay	Comparison	Median OS (months) (hazard ratio, 95% confidence interval)	Median PFS (months) (hazard ratio, 95% confidence interval)	Other outcome measures if OS and PFS not reported
*Lung cancer*					
IMpower 131 [82]	SP142	Atezolizumab + carboplatin + nab-paclitaxel vs carboplatin + nab-paclitaxel	ITT: 14.2 vs 13.5 (0.88, 0.73–1.05) TC3 or IC3: 23.4 vs 10.2 (0.48, 0.29–0.81)^a^	ITT: 6.3 vs 5.6 (0.71, 0.60–0.85)^a^ TC3 or IC3: 10.1 vs 5.1 (0.41, 0.25–0.68)^a^	
IMpower150 [83]	SP142	Atezolizumab + carboplatin + paclitaxel vs bevacizumab + carboplatin + paclitaxel	ITT: 19.0 vs 14.7 (0.84, 0.71–1.00) TC3 or IC3: 26.3 vs 15.0 (0.76, 0.49–1.17) TC1/2/3 or IC1/2/3: 24.4 vs 16.0 (0.71, 0.55–0.91)^a^ TC0 and IC0: 14.8 vs 14.1 (0.96, 0.76–1.22)	ITT: 6.3 vs 6.8 (0.82, 0.70–0.97)^a^ TC3 or IC3: 8.3 vs 6.8 (0.57, 0.39–0.83)^a^ TC1/2/3 or IC1/2/3: 7.1 vs 6.8 (0.67, 0.53–0.83)^a^ TC0 and IC0: 5.4 vs 6.9 (0.98, 0.78–1.23)	
		Atezolizumab + bevacizumab + carboplatin + paclitaxel vs bevacizumab + carboplatin + paclitaxel	ITT: 19.5 vs 14.7 (0.80, 0.67–0.95)^a^ TC3 or IC3: 30.0 vs 15.0 (0.70, 0.46–1.08) TC1/2/3 or IC1/2/3: 22.5 vs 16.0 (0.73, 0.57–0.94)^a^ TC0 and IC0: 16.9 vs 14.1 (0.90, 0.71–1.14)	ITT: 8.4 vs 6.8 (0.57, 0.48–0.67)^a^ TC3 or IC3: 15.2 vs 6.8 (0.34, 0.23–0.50)^a^ TC1/2/3 or IC1/2/3: 11.1 vs 6.8 (0.47, 0.38–0.60)^a^ TC0 and IC0: 7.2 vs 6.9 (0.71, 0.57–0.89)^a^	
KEYNOTE-189 [84]	22C3	Pembrolizumab + pemetrexed-platinum vs placebo + pemetrexed-platinum	ITT: 22.0 vs 10.7 (0.56, 0.45–0.70)^a^ TPS ≥ 50%: not reached vs 10.1 (0.59, 0.39–0.88)^a^ TPS 1-49%: 21.8 vs 12.1 (0.62, 0.42–0.92)^a^ TPS < 1%: 17.2 vs 10.2 (0.52, 0.36–0.74)^a^	ITT: 9.0 vs 4.9 (0.48, 0.40–0.58)^a^ TPS ≥ 50%: 11.1 vs 4.8 (0.36, 0.26–0.51)^a^ TPS 1%–49%: 9.2 vs 4.9 (0.51, 0.36–0.73)^a^ TPS < 1%: 6.2 vs 5.1 (0.64, 0.47–0.89)^a^	
IMpower133 [85]	SP263	Atezolizumab + carboplatin + etoposide vs placebo + carboplatin + etoposide	ITT: 12.3 vs 10.3 (0.76, 0.60–0.95)^a^ ≥5% TC or IC: 21.6 vs 9.2 (0.60, 0.25–1.46) <5% TC or IC: 9.2 vs 8.9 (0.77, 0.51–1.17) ≥1% TC or IC: 9.7 vs 10.6 (0.87, 0.51–1.49) <1% TC and IC: 10.2 vs 8.3 (0.51, 0.30–0.89)^a^	ITT: 5.2 vs 4.3 (0.77, 0.62–0.96)^a^ ≥1% TC or IC: 5.1 vs 5.5 (0.86, 0.51–1.46) <1% TC or IC: 5.4 vs 4.2 (0.52, 0.31–0.88)^a^	
IMpower132 [86]	SP142	Atezolizumab + carboplatin or cisplatin + pemetrexed vs carboplatin or cisplatin + permetrexed	ITT: 17.5 vs 13.6 (0.86, 0.71–1.06) TC3 or IC3: not reached vs 26.9 (0.73, 0.31–1.73) TC1/2 or IC1/2: 12.7 vs 16.2 (1.18, 0.80–1.76) TC0 and IC0: 15.9 vs 10.5 (0.67, 0.46–0.96)^a^	ITT: 7.7 vs 5.2 (0.56, 0.47–0.67)^a^ TC3 or IC3: 10.8 vs 6.5 (0.46, 0.22–0.96)^a^ TC1/2 or IC1/2: 6.2 vs 5.7 (0.80, 0.56–1.16) TC0 and IC0: 8.5 vs 4.9 (0.45, 0.31–0.64)^a^	
IMpower110 [87]	SP142	Atezolizumab vs chemotherapy	TC3 or IC3: 20.2 vs 13.1 (0.59, 0.40–0.89)^a^ TC2/3 or IC2/3: 18.2 vs 14.9 (0.72, 0.52–0.99)^a^ TC1/2/3 or IC1/2/3: 17.5 vs 14.1 (0.83, 0.65–1.07)	TC3 or IC3: 8.1 vs 5.0 (0.63, 0.45–0.88)^a^ TC2/3 or IC2/3: 7.2 vs 5.5 (0.67, 0.52–0.88)^a^ TC1/2/3 or IC1/2/3: 5.7 vs 5.5 (0.77, 0.63–0.94)^a^	
IMpower130 [88]	SP142	Atezolizumab + carboplatin + nab-paclitaxel vs carboplatin + nab-paclitaxel	ITT: 18.6 vs 13.9 (0.79, 0.64–0.98)^a^ TC3 or IC3: 17.3 vs 16.9 (0.84, 0.51–1.39) TC1/2 or IC1/2: 23.7 vs 15.9 (0.70, 0.45–1.08) TC0 and IC0: 15.2 vs 12.0 (0.81, 0.61–1.08)	ITT: 7.0 vs 5.5 (0.64, 0.54–0.77)^a^ TC3 or IC3: 6.4 vs 4.6 (0.51, 0.34–0.77)^a^ TC1/2 or IC1/2: 8.3 vs 6.0 (0.61, 0.43–0.85)^a^ TC0 and IC0: 6.2 vs 4.7 (0.72, 0.56–0.91)^a^	
KEYNOTE-010 [89]	22C3	Pembrolizumab 2 mg/kg vs docetaxel	ITT: 10.4 vs 8.5 (0.71, 0.58–0.88)^a^ ≥50% TC: 14.9 vs 8.2 (0.54, 0.38–0.77)^a^	ITT: 3.9 vs 4.0 (0.88, 0.74–1.05) ≥50% TC: 5.0 vs 4.1 (0.59, 0.44–0.78)^a^	
		Pembrolizumab 10 mg/kg vs docetaxel	ITT: 12.7 vs 8.5 (0.61, 0.49–0.75)^a^ ≥50% TC: 17.3 vs 8.2 (0.50, 0.36–0.70)^a^	ITT: 4.0 vs 4.0 (0.79, 0.66–0.94)^a^ ≥50% TC: 5.2 vs 4.1 (0.59, 0.45–0.78)^a^	
KEYNOTE-042 [90]	22C3	Pembrolizumab vs investigator’s choice of platinum-based chemotherapy	TPS ≥ 50%: 20.0 vs 12.2 (0.69, 0.56–0.85)^a^ TPS ≥ 20%: 17.7 vs 13.0 (0.77, 0.64–0.92)^a^ TPS ≥ 1%: 16.7 vs 12.1 (0.81, 0.71–0.93)^a^	TPS ≥ 50%: 7.1 vs 6.4 (0.81, 0.67–0.99)^a^ TPS ≥ 20%: 6.2 vs 6.6 (0.94, 0.80–1.11) TPS ≥ 1%: 5.4 vs 6.5 (1.07, 0.94–1.21)	
KEYNOTE-407 [91]	22C3	Pembrolizumab + carboplatin + paclitaxel or nab-paclitaxel vs placebo + carboplatin + paclitaxel or nab-paclitaxel	ITT: 15.9 vs 11.3 (0.64, 0.49–0.85)^a^ TPS ≥ 50%: not reached in either arm (0.64, 0.37–1.10) TPS 1–49%: 14.0 vs 11.6 (0.57, 0.36–0.90)^a^ TPS < 1%: 15.9 vs 10.2 (0.61, 0.38–0.98)^a^	ITT: 6.4 vs 4.8 (0.56, 0.45–0.70)^a^ TPS ≥ 50%: 8.0 vs 4.2 (0.37, 0.24–0.58)^a^ TPS 1–49%: 7.2 vs 5.2 months (0.56, 0.39–0.80)^a^ TPS < 1%: 6.3 vs 5.3 (0.68, 0.47–0.98)^a^	
OAK [92]	SP142	Atezolizumab vs docetaxel	ITT: 13.8 vs 9.6 (0.73, 0.62–0.87)^a^ TC3 or IC3: 20.5 vs 8.9 (0.41, 0.27–0.64)^a^ TC2/3 or IC2/3: 16.3 vs 10.8 (0.67, 0.49–0.90)^a^ TC1/2/3 or IC1/2/3: 15.7 vs 10.3 (0.74, 0.58–0.93)^a^ TC0 and IC0: 12.6 vs 8.9 (0.75, 0.59–0.96)^a^	ITT: 2.8 vs 4.0 (0.95, 0.82–1.10) TC3 or IC3: 4.2 vs 3.3 (0.63, 0.43–0.91)^a^ TC2/3 or IC2/3: 4.1 vs 3.6 (0.76, 0.58–0.99)^a^ TC1/2/3 or IC1/2/3: 2.8 vs 4.1 (0.91, 0.74–1.12) TC0 and IC0: 2.6 vs 4.0 (1.00, 0.80–1.25)	
ONO-4538-52/TASUKI-52 [93]	28-8	Nivolumab + carboplatin + paclitaxel + bevacizumab vs placebo + carboplatin + paclitaxel + bevacizumab	ITT: 25.4 vs 24.7 (0.85, 0.63–1.14)	ITT: 12.1 vs 8.1 (0.56, 0.43–0.71)^a^ ≥50% TC: 9.9 vs 6.9 (0.55, 0.36–0.83)^a^ 1–49% TC: 11.0 vs 8.4 (0.63, 0.42–0.96)^a^ <1% TC or indeterminate: 13.6 vs 8.4 (0.55, 0.38–0.78)^a^	
*Melanoma*					
CheckMate 238 [94]	28-8	Nivolumab vs ipilimumab			4-year recurrence-free survival rate ITT: 51.7 vs 41.2 (0.71, 0.60–0.86)^a^ ≥5% TC: 64.0% vs 52.3% (0.67, 0.47–0.96)^a^ <5% TC: 44.2% vs 35.7% (0.75, 0.60–0.93)^a^ ≥1% TC: 56.4% vs 45.2% (0.68, 0.54–0.86)^a^ <1% TC: 40.6% vs 33.2% (0.79, 0.58–1.08)
*Bladder cancer*					
JAVELIN Bladder 100 [95]	SP263	Avelumab + best supportive care vs best supportive care	ITT: 21.4 vs 14.3 (0.69, 0.56–0.86)^a^ PD-L1 positive (≥25% TC, or ≥25% IC if >1% tumour area contains IC, or 100% IC if <1% tumour area contains IC): not reached vs 17.1 (0.56, 0.40–0.79)^a^ PD-L1 negative (criteria for PD-L1 positivity not met): 18.8 vs 13.7 (0.85, 0.62–1.18)	ITT: 3.7 vs 2.0 (0.62, 0.52–0.75)^a^ PD-L1 positive: 5.7 vs 2.1 (0.56, 0.43–0.73)^a^ PD-L1 negative: 3.0 vs 1.9 (0.63, 0.47–0.85)^a^	
IMvigor010 [96]	SP142	Atezolizumab vs observation			Median disease-free survival ITT: 19.4 vs 16.6 (0.89, 0.74–1.08) IC0/1: 16.4 vs 11.1 (0.85, 0.66–1.10) IC2/3: 24.8 vs 41.4 (1.01, 0.76–1.35)
CheckMate 274 [97]	28-8	Nivolumab vs placebo			6-month disease-free survival ITT: 74.9% vs 60.3% (0.70, 0.55–0.90)^a^ ≥1% TC: 74.5% vs 55.7% (0.55, 0.35–0.85)^a^ 6-month recurrence-free survival ITT: 77.0% vs 62.7% (0.72, 0.59–0.89)^a^ ≥1% TC: 75.3% vs 56.7% (0.55, 0.39–0.79)^a^
KEYNOTE-361 [98]	22C3	Pembrolizumab vs gemcitabine + investigator’s choice of cisplatin or carboplatin	ITT: 15.6 vs 14.3 (0.92, 0.77–1.11) CPS ≥ 10: 16.1 vs 15.2 (1.01, 0.77–1.32)		

A search using the term “PD-L1” was conducted in the PubMed database and all Phase III trials in lung, melanoma and bladder cancer which stratified patients by PD-L1 expression were included. Included trials stated use of an FDA-approved antibody assay (22-8, 22C3, SP263 or SP142) and reported both raw data and hazard ratios with a 95% confidence interval. Trials testing a combination of a PD-1 pathway inhibitor with another drug were excluded.

*OS* overall survival, *PFS* progression-free survival, *ITT* intention to treat, *TC* tumour cell, *IC* immune cell.

^a^Statistically significant.

TC0, TC1, TC2 and TC3 refer to PD-L1 expression on <1%, ≥1% and <5%, ≥5% and <50% and ≥50% tumour cells, respectively.

IC0, IC1, IC2 and IC3 refer to PD-L1 expression on <1%, ≥1% and <5%, ≥5% and <50% and ≥50% immune cells, respectively.

TPS: percentage of tumour cells showing staining for PD-L1.

CPS: percentage of tumour and immune cells showing staining for PD-L1.

While finding the optimal PD-L1 expression cut-off may be difficult, another difficulty arises from the fact that it may not be possible to establish a single standardised threshold suitable for all cancer types; a study analysing all primary studies associated with FDA approvals of ICIs found that the PD-L1 threshold varied not only between cancers but also within cancer types [22]. Therefore, suitable PD-L1 cut-offs may need to be determined on a cancer subtype-by-subtype basis. Further complicating the matter is the presence of variation in PD-L1 expression between the primary tumour and metastases, as well as between metastases [23–27]. This means that taking a measurement from one tumour may not give an accurate representation of the PD-L1 status of other tumours in the body. In addition to intertumoural differences in PD-L1 expression, there may also be intratumoural heterogeneity [28, 29]. Consequently, multiple biopsies from the tumour, and biopsies from several metastases, would be required to more accurately determine PD-L1 status.

### Inflammatory tumour microenvironment

Although PD-L1 expression may indicate that other features of an inflammatory tumour microenvironment (TME) such as abundant T cells and IFN-γ signalling are present, the two are not always correlated. PD-L1 expression can be upregulated through one of two mechanisms: innate or adaptive immune resistance. Innate immune resistance refers to PD-L1 upregulation by oncogenic pathways that are intrinsic to the tumour, for instance, the oncogenic kinase NPM/ALK, often seen in anaplastic large-cell lymphoma, induces PD-L1 upregulation via the transcription factor STAT3 [30]. Innate immune resistance is associated with a ‘cold’ or ‘immune-desert’ TME in which tumours exhibit high PD-L1 expression but low TIL density. In contrast, adaptive immune resistance occurs when PD-L1 is upregulated by pro-inflammatory cytokines such as IFN-γ released from activated T cells and is associated with a ‘hot’ or ‘immune-inflamed’ TME. These tumours show high PD-L1 expression, TIL abundance and IFN-γ signaling [31]. The hot TME seen in adaptive immune resistance, but not the cold TME seen in innate immune resistance, is associated with response to PD-1 pathway inhibition [32].

Hot and cold TMEs cannot be distinguished based on PD-L1 status alone and instead require measurement of TIL abundance and IFN-γ signalling. The presence of CD8^+^ TILs in the TME can be determined through immunohistochemical techniques and this has been shown to be associated with response to immune checkpoint blockade in metastatic melanoma patients [33]. IFN-γ signalling on the other hand can be determined by analysing the gene expression profiles of tumour cells and TILs. One study found that an IFN-γ-related mRNA profile in TILs was correlated with response to PD-1 inhibition [34], while a separate study found that high baseline levels of tumoural IFN-γ mRNA were associated with the higher response rate in durvalumab-treated NSCLC patients [35].

Direct measurements of TIL abundance and IFN-γ signalling require tumour biopsies to be taken; however, the inflammatory TME has also been linked to higher levels of inflammatory cytokines in the peripheral blood—a study found that responders to nivolumab had higher baseline IFN-γ and IL-6 levels in the serum. Responders also had higher levels of the anti-inflammatory cytokine IL-10, which likely represented a simultaneous suppression of the anti-tumour response [36]. In addition to an increase in peripheral cytokines, responders may also show increased levels of peripheral CD14^+^CD16^−^ monocytes expressing higher levels of the migration and activation markers ICAM-1 and HLA-DR [37]. This reflects a stronger baseline anti-tumour response, as monocytes are formed from IFN-γ-stimulated myeloid-biased haematopoietic stem cell differentiation [38]. Importantly, these monocytes are distinct from myeloid-derived suppressor cells (CD33^lo^CD11b^+^HLA-DR^lo^ myeloid cells) which counteract the host anti-tumour response and are associated with poor response to PD-1 blockade [39]. Together, these studies demonstrate that it is possible to gain information about the anti-tumour response from peripheral markers, and in practice, such biomarkers may be useful in confirming biopsy results where there is ITH or multiple metastases.

Most studies examining TILs in the TME have focused on CD8^+^ T cells, however recently it has been shown that the abundance of B cells within the tumour is also an excellent predictor of response. B cells play a complex role in tumour pathology; they can bring about antibody-dependent cell death, present tumour antigens to T cells, and form plasmablast-like cells that secrete T-cell-recruiting chemokines; however, they also release inhibitory factors that limit the anti-tumour immune response. Despite their complex role, studies have found that B cell markers including CD19 and CD20, detected either histologically or by RNA sequencing, in human tumour immune cell infiltrates are associated with response to ICIs [40, 41], with one study finding that these B cell markers were the most differentially expressed genes between responders and non-responders [42]. Histological and transcriptomic studies suggest that these B cells exist within tertiary lymphoid structures (TLSs) containing T cells, follicular dendritic cells and B cells, and that the presence of TLSs themselves predict higher survival and response rate to PD-1 blockade [43, 44]. A recent study proposed that the B cell response to tumour cells may be modified by the cytokine LIF. Using a proteomics approach, the study showed that high plasma levels of LIF were associated with the absence of TLSs and a poor clinical outcome in patients treated with PD-1 pathway blockade, independent of other prognostic factors [45]. These studies show that the B cell anti-tumour response is a promising biomarker warranting further investigation.

Aside from lymphocytes, myeloid cells may also play an important role in the TME. One study identified an abundance of myeloid cells expressing CXCL9, a cytokine induced by IFN-γ, in responders to atezolizumab and avelumab [46]. An analysis of clinical trial datasets for avelumab and atezolizumab showed a 2.4- and 2.8-fold increase in overall survival respectively for patients with the highest CXCL-9 expression compared to the lowest, which may be due to the CXCL9-CXCR3 axis promoting PD-L1 expression and increasing T cell recruitment [47–49]. This suggests a role for the analysis of other immune cells in the TME besides lymphocytes.

In addition to the direct detection of immune cells within the TME, mutations that indirectly alter the tumour immune microenvironment have also been identified as potential predictors of response. One study examined three distinct subtypes of *KRAS*-driven lung adenocarcinoma; the KL subtype which in addition to the *KRAS* mutation also has a comutation in *STK11*/*LKB1*, the KP subtype which has a comutation in *TP53*, and the K-only subtype which does not have a comutation in either of the tumour suppressor genes *STK11*/*LKB1* or *TP53*. Out of these three subtypes, it was found that the KL subtype was associated with reduced objective response rate and progression-free survival following anti-PD-1 treatment [50]. This is likely due to the tendency of *STK11/LKB1* inactivation to result in a ‘cold’ TME with reduced TIL abundance and activity [51, 52]. The KP subtype on the other hand tended to have an increased disease control rate, due to its association with a high TMB, IFN-γ and PD-L1 expression [53]. A more recent study revealed that within the KP subtype, different mutations in *TP53* were associated with different levels of response to PD-1 pathway blockade; while missense *TP53* mutations were associated with increased PD-L1 expression and IFN-γ signatures and had a greater response to PD-1 pathway blockade, nonsense *TP53* mutations were associated with enrichment of immune suppressor cells such as M2 macrophages and neutrophils and were not associated with improved response [54].

*JAK1* and *JAK2* mutations have also been implicated in affecting the tumour immune microenvironment. A study in patients who had initially responded to pembrolizumab and subsequently relapsed used whole-exome sequencing to identify mutations that caused resistance to therapy and found that *JAK1* and *JAK2* loss of function mutations with deletion of the wildtype allele was associated with resistance due to an inability of JAK1 and JAK2 deficient tumours to respond to IFN-γ stimulation by expressing proteins involved in antigen presentation and suppressing their own growth [55]. This study identified these mutations as a cause of developed resistance; however, a separate smaller study found that *JAK1*/*2* mutations were present in pre-treatment tumour biopsies from two patients with a high TMB but poor response to anti-PD-1 therapy, suggesting these mutations may also be a primary cause of resistance to anti-PD-1 therapy [56].

### PD-L1 expression on tumour infiltrating immune cells

The traditional view that PD-1 blockade primarily interrupts signaling at tumour cell PD-L1 has led to a focus on studying PD-L1 expression on tumour cells as a biomarker, to the neglect of PD-L1 expression on immune cells such as lymphocytes, macrophages and dendritic cells. The predictive value of PD-L1 expression on tumour infiltrating immune cells (TIIs) could explain the consistent finding that some patients with PD-L1 negative tumours still benefit from PD-1 pathway blockade, an effect which is especially pronounced in melanomas [57]. One study found that a third of patients with PD-L1-negative melanomas (cut-off of <5%) and melanomas with indeterminate PD-L1 expression responded to nivolumab [58]. This could be due to the presence of PD-L1-expressing antigen-presenting cells (APCs) in lymphoid tissue and PD-L1-expressing TIIs within the tumour.

Although one study showed that tumour cell PD-L1 was predictive of response across multiple cancer types while PD-L1 expression on TIIs did not reach statistical significance [17], other studies have shown that TII PD-L1 expression is more predictive of response than tumour PD-L1 expression [59–62], demonstrating the need for more research into TII PD-L1 expression. A possible explanation for these conflicting findings is that the predictive values of tumour and TII PD-L1 expression depends on the drug target; the response to nivolumab (an anti-PD-1 antibody) appears to correlate with PD-L1 expression on tumour cells but not TIIs, while the response to atezolizumab and avelumab (anti-PD-L1 antibodies) correlates with PD-L1 expression on TIIs. However, much more research is required to draw any definitive conclusions about this.

## Biomarkers in non-tumour samples

### HLA heterozygosity and mutations affecting MHC-I

Before tumour neoantigens can be recognised by CD8^+^ T cells, they must first be presented on the tumour cell surface using MHC-I molecules. Factors affecting the ability of MHC-I to present tumour neoantigens will therefore impact the response to PD-1 pathway blockade. MHC-I molecules consist of a non-polymorphic region encoded by the β-2 microglobulin gene (*B2M*) and a highly polymorphic region encoded by the human leukocyte antigen (HLA) class I genes at the *HLA-A*, *HLA-B* and *HLA-C* loci.

One study found that maximal heterozygosity at the HLA class I loci is associated with increased overall survival after ICI therapy, as the presence of a larger number of types of HLA class I molecules allows a greater range of tumour neoantigens to be presented to CD8^+^ T cells [63]. The same study also observed greater T cell receptor clonality during therapy in patients with HLA heterozygosity, suggesting HLA heterozygosity may improve clonal selection and expansion of T cells. Truncating mutations in *B2M* have also been linked to reduced response to ICI therapy, as this disrupts the ability of the MHC molecule to be expressed on the cell surface [55]. However, unlike HLA heterozygosity, these mutations are seen as de novo mutations in tumour cells, rather than as host germline mutations. *B2M* mutations and other mutations resulting in MHC-I downregulation are seen across many cancer types, including colorectal cancer, bladder cancer and breast cancer, as a mechanism to facilitate immune evasion [64–66].

A final point to consider is that different HLA alleles vary in the size of their binding repertoire and their binding strength. A study analysing the immunogenicity of peptides of varying binding affinity in transgenic mice expressing wide (A*0201), intermediate (B*0702) or narrow (A*0101) repertoires demonstrated that alleles with a wider repertoire and higher binding affinity were associated with a greater immune response, and that use of allele-specific affinity thresholds allowed better prediction of immunogenicity [67]. Taken together, the aforementioned studies show that factors contributing to host MHC-I function, including HLA heterozygosity, expression on the cell surface, repertoire size and peptide binding strength, are all important determinants of immune response generation.

### Autoimmune genetic susceptibility

The therapeutic effect of PD-1 pathway blockade relies on the ability of host T cells to recognise tumour antigens. Tumour antigens may arise from missense mutations in normal genes and therefore they may differ only subtly from self-antigens. The PD-1 pathway itself is also involved in the prevention of autoimmunity. As a result, host susceptibility to autoimmune disease may be a predicting factor for a response. A study in 436 patients with metastatic melanoma found that rs17388568, a single nucleotide polymorphism in the *IL-2*/*IL-21* locus that increases the risk of allergy, colitis and type 1 diabetes, was associated with improved response to anti-PD-1 therapy [68]. In addition, around 50% of patients with the pre-existing autoimmune disease develop a flare of their pre-existing disease upon ICI treatment [69]. This may account for a significant proportion of total irAEs, with one study finding that out of 35 patients experiencing irAEs due to pembrolizumab or nivolumab, 8 of these were flares of pre-existing autoimmune disease [70]. While autoimmune disease is linked to irAE occurrence, irAE occurrence has in turn been linked to ICI response, for instance an association has been demonstrated between irAEs and recurrence-free survival in melanoma patients treated with pembrolizumab [71], as well as between the severity of skin toxicity and increased progression-free survival and overall survival following ICI treatment [72]. These studies provide further support for the role of autoimmunity in the response to PD-1 pathway blockade. However, the limited number of studies directly analysing the link between autoimmunity and response to PD-1 pathway blockade means that more research is required before a definitive link can be made.

### Gut microbiota composition

The gut microbiota is known to affect the host immune system and studies suggest that the microbiota of responders to PD-1 pathway blockade differs from that of non-responders. A recent meta-analysis showed that responders and non-responders do not have significant differences in diversity, suggesting any differences are due to specific bacterial species present [73]. This meta-analysis identified 17 species differentially abundant in responders compared with non-responders, which importantly did not cluster by the study of origin. The most abundant species present in responders were an unknown *Ruminococcaceae* species, unknown *Faecalibacterium* species, *Ruminococcus bicirculans*, and *Barnesiella intestinihominis*, whereas non-responders tended to be enriched in *Bacteroides thetaiotaomicron*, *Adlercreutzia equolifaciens*, *Bifidobacterium dentium* and unknown *Mogibacterium*. However, this meta-analysis was limited to patients with metastatic melanoma and therefore the findings may not be applicable to the wider population of cancer patients who may express a different set of tumour antigens on their tumour cells and therefore lack any molecular mimicry between tumour cells and gut bacteria. Several separate studies have found various new associations including abundant *Prevotella*, *Lachnospiraceae* and *Faecalibacterium prausnitzii*, among several other species, in anti-PD-1 and anti-PD-L1 responders, and a greater *Bacteroidales* abundance in non-responders [74–76]. However, a major limitation of studies of the microbiota is that the simultaneous analysis of a large number of different species within the gut may result in the detection of spurious correlations. There is consequently a major role for meta-analysis to play in identifying true associations between microbiota and response to PD-1 pathway blockade.

Several mechanisms have been proposed through which the gut microbiome may modulate host immunity. First, molecular mimicry may occur between the antigens of gut microorganisms and tumour antigens, stimulating a stronger immune response against tumour cells [77]. Another potential mechanism is that gut microorganisms might modulate the tumour immune microenvironment – in anti-PD-1 responders, a significant correlation was found between baseline CD8^+^ T cells in the TME and the abundance of *Faecalibacterium* genus, *Ruminococcaceae* family and *Clostridiales* order, suggesting these microorganisms may promote the development of a hot TME, whereas a negative association was seen with *Bacteroidales* [78]. When looking at host immunity at a systemic level in the same patient cohort, it was found that patients with abundant *Faecalibacterium*, *Ruminococcaceae* and *Clostridiales* had increased systemic circulation levels of CD4^+^ and CD8^+^ T cells and a greater cytokine response to anti-PD-1, whereas those with abundant *Clostridiales* had higher levels of regulatory T cells and myeloid-derived suppressor cells and a reduced cytokine response to anti-PD-1, suggesting that the gut microbiota was not only capable of modulating the host anti-tumour response at the TME level, but also at the systemic level. The distant effects of the gut microbiota may arise due to gut microorganisms directly stimulating local innate immune cells which then travel distantly to the tumour site. Alternatively, escaped microbial products in the lymph nodes and blood may stimulate cytokine release distantly, contributing to improved immunosurveillance [77].

## Conclusion

Currently, PD-L1 expression, high TMB and MMR deficiency stand as the most robust predictive biomarkers of response to PD-1 pathway inhibition and have been approved for clinical use. However, these biomarkers are limited by ITH and the lack of a standardised threshold. Furthermore, they do not perfectly correlate with response rate and so none of them can be used with high predictive accuracy in isolation. Growing evidence for other potential biomarkers may allow clinical response to be more accurately predicted. This review addresses each biomarker in turn; however, it is important to acknowledge that the different biomarkers interact with each other as part of a balance between the pro-inflammatory anti-tumour immune response and the immune evasion mechanisms used by the tumour to suppress this response. To illustrate, MMR deficiency and certain mutations can lead to a high TMB, which in turn increases the chances of forming immunogenic neoantigens. The ability of the host immune system to recognise these neoantigens at the level of TCR ligation is affected by factors such as HLA heterozygosity and gut microbiota molecular mimicry, while the ability of T cells to destroy the tumour cells is dependent on the level of immune infiltration into the tumour. These factors all contribute to the host anti-tumour response, however IFN-γ secreted by activated T cells in the process can stimulate PD-L1 expression on tumour cells and TIIs, resulting in immune evasion. The interlinking nature of these biomarkers suggests that they should not be viewed in isolation but should be considered as part of a larger picture of the balance between immune response and immune evasion.

To assess the full range of potential biomarkers, this may involve taking multiple biopsies from the tumour to test for biomarkers within the TME while accounting for ITH, a blood sample to analyse peripheral biomarkers and host germline genetics, and a stool sample to assess the gut microbiota. From each of these samples, a combination of genetic and non-genetic tests would then enable the detection of each biomarker. For instance, through genetic tests on the tumour biopsy, TMB can be assessed using targeted cancer gene sequencing, tumour neoantigen immunogenicity can be determined using transcriptome sequencing, and MMR deficiency can be detected using MSI-PCR. Using immunohistochemical techniques tests on the tumour biopsy, tumour and TII PD-L1 expression can be assessed and TIL abundance can be quantified. Using genetic techniques on a peripheral blood sample, HLA heterozygosity and specific mutations that affect MHC-I can be detected with DNA sequencing, and autoimmune genetic susceptibility can be determined with single nucleotide polymorphism genotyping. Finally, 16 S rRNA sequencing of microorganisms in the stool sample would allow characterisation of the gut microbiota. Taken together, these results would provide a thorough assessment of the proposed biomarkers.

However, such tests would be limited by the practicalities of cost, time, and resources. Therefore, more research is needed to identify the biomarkers with the greatest predictive value. One bioinformatic study analysing biomarkers from large clinical datasets identified 55 candidate biomarkers predicting response to ICI therapy, including clonal TMB, total TMB and expression of CXCL9 [79]. When the 11 strongest markers were used to stratify patients, this more accurately distinguished responders from non-responders than TMB alone. This was also the case, although to a lesser extent, when a simplified 2-marker model comprising clonal TMB and CXCL9 expression was used, suggesting a role for the use of a few biomarkers with high predictive value, as opposed to a single biomarker or a full assessment of all biomarkers. This method of biomarker assessment would be both time-efficient and cost-effective, as it allows accurate response prediction with minimal testing, and should perhaps be the ultimate goal of biomarker research. However, we are still a long way from employing this in clinical practice; it would require an understanding, and evidence-base, that we have not yet built.

Although not discussed in detail in this review, it is important to note that some biomarkers are modifiable, generating the possibility of priming a patient to ensure they have the highest chances of response before therapy is initiated. For instance, therapeutic response to PD-1 pathway blockade can be enhanced in mice by modifying the gut microbiota through faecal microbiota transplantation [78] or oral administration of bacteria [80]. Another modifiable factor is the tumour immune microenvironment, which can be converted from cold to hot using certain chemotherapeutic drugs—combined treatment of murine lung adenocarcinoma models using oxaliplatin and cyclophosphamide was found to be associated with an increased CD8^+^:regulatory T cell ratio within tumours, increased presence of tumour-antigen-specific TILs, increased TIL PD-1 expression, and increased PD-L1 expression by tumour stromal cells [81]. These results are yet to be shown in humans; however, they do illustrate the possibility of modifying host factors to optimise the therapeutic effect. The field of biomarker development is hugely promising and, as medicine becomes increasingly personalised and as we learn more about biomarkers, they will inevitably play a large role in informing treatment decisions.

## Data Availability

This article does not contain newly created or analysed data.
